# Bacterial corneal ulcer associated with common variable immune deficiency

**DOI:** 10.1186/s12348-016-0098-8

**Published:** 2016-08-05

**Authors:** Edmund Tsui, Jie Deng, Andrew N Siedlecki, Michael E Zegans

**Affiliations:** 1 Department of Ophthalmology, New York University School of Medicine, New York, NY USA; 2 Department of Surgery, Dartmouth-Hitchcock Medical Center, Lebanon, NH USA; 3 Geisel School of Medicine at Dartmouth, Hanover, NH USA; 4 Section of Ophthalmology, Dartmouth-Hitchcock Medical Center, 1 Medical Center Drive, Lebanon, NH 03756 USA

**Keywords:** Common variable immunodeficiency, Bacterial keratitis, Autoimmune disease, Immunosuppression

## Abstract

Common variable immune deficiency (CVID) is one of the most commonly diagnosed primary immunodeficiencies. Generally, patients have a history of recurrent sinopulmonary infections, hypogammaglobulinemia of two or more immunoglobulin isotypes, and impaired functional antibody responses. Reports of corneal involvement associated with CVID are limited. We describe a case of corneal ulceration associated with methicillin-resistant *Staphylococcus aureus* in a patient with CVID that developed while on monthly intravenous immunoglobulin infusions and in which there were no common risk factors for bacterial keratitis, such as prior history of ocular surface disease, trichiasis, trauma, or contact lens wear.

## Introduction

Common variable immune deficiency (CVID) is the most commonly encountered symptomatic primary immunodeficiency [1]. Herein, we describe an unusual presentation of a corneal ulcer in a patient with a history of CVID.

## Case report

An 82-year-old female who was routinely followed for bilateral dry age-related macular degeneration (AMD) was noted to have corneal thinning in her left eye and referred to the cornea service for further evaluation. The patient had a history of hypogammaglobulinemia diagnosed approximately 8.5 years prior to presentation with a documented history of low immunoglobulin levels, specifically an IgG of 61 mg/dL, IgA < 16 mg/dL, and IgM < 5 mg/dL. At that time, she began monthly intravenous immunoglobulin (IVIG) infusions. Past ocular history was pertinent for bilateral cataract extraction with intraocular lens implant 15 years prior to presentation and dry AMD bilaterally. There was no history of ocular trauma, lagophthalmos, or contact lens wear. She had a history of recurrent sinus infections since her teenage years. Additionally, she had a history of oral and esophageal lichen planus treated with oral cyclosporine, recurrent urinary tract infections, microscopic colitis, diabetes mellitus, and hypertension. On initial presentation, she complained of irritation of the left eye for 2 weeks. The visual acuity was 20/20 in her right eye at distance, and her left eye was 8/200e at distance and 20/800 at near. There was no afferent pupillary defect in her right eye, but her left eye was unable to be assessed due to significant corneal opacities. Intraocular pressures were 10 mmHg in the right eye and 9 mmHg in the left eye. On slit lamp exam of her left eye, there were dilated iris vessels in all quadrants and a 1.0 mm × 1.0 mm (1.00 mm^2^) area of minimal thinning of the central cornea with intact epithelium without staining or infiltrate. Funduscopic exam was unchanged from prior examinations, demonstrating extensive macular pigment atrophy bilaterally without hemorrhage. Given the sterile appearance, she was started on Tobradex (tobramycin/dexamethasone) ophthalmic ointment four times daily. She was evaluated by the retina service 6 days after initial presentation with an unchanged funduscopic exam without evidence of ocular ischemia and a stable corneal exam. Upon follow-up 2 weeks after initial presentation, her visual acuity in her left eye was 4/200e at distance and 20/800 at near, there was 2+ conjunctival injection, there were dilated iris vessels, and her left cornea had a central 1.5-mm area of 90 % thinning with epithelial defect but still no infiltrate. Tobradex was stopped, she was started on doxycycline 100 mg twice daily and Vigamox four times daily, and corneal cultures were obtained. Clinical labs were negative, including c-ANCA, p-ANCA, ANA, myeloperoxidase antibody, PR-3 antibody, syphilis IgG, and rheumatoid factor level of 10 units. Cultures from her left cornea grew methicillin-resistant *Staphylococcus aureus* (MRSA) within 48 h. Vancomycin 25 mg/cc drops was added every 2 h, and oral doxycycline and Vigamox were continued. She was seen regularly for follow-up with eventual healing of her epithelial defect over the following month. Two months after initial presentation, she developed an acute conjunctivitis in her left eye. Conjunctival cultures were obtained, and she was started empirically on ciprofloxacin ophthalmic ointment four times daily. Conjunctival cultures eventually grew MRSA, so vancomycin 25 mg/cc drops four times daily and oral doxycycline 100 mg twice daily were restarted, and she continued her topical ciprofloxacin ophthalmic ointment four times daily. At follow-up 2.5 months after initial presentation, her conjunctivitis had resolved and corneal thinning had been stable at 70–80 % thinning, but iris vessels of the left eye remained significantly dilated. She was started on Tobradex ophthalmic ointment twice daily (given that her prior MRSA cultures were gentamicin sensitive and to reduce the inflammation likely driving her persistently dilated left eye iris vessels) and continued her oral doxycycline 100 mg twice daily. Four months after initial presentation, her left cornea had a central 2.0-mm area of 80 % corneal thinning and scarring but with no infiltrate or epithelial defect and a left eye visual acuity at 20/400. At her most recent appointment, 8 months after the ulcer was initially noted, her left eye visual acuity was 20/400 and her left cornea had a central 2.0-mm area of 80 % corneal thinning with irregularities of the epithelium but no epithelial infiltrates or defect. She remains on artificial tears four times daily. Her vision in her left eye remains limited by her AMD, with visual acuity prior to the episode of corneal ulcer ranging from 20/400 to 20/800.

## Discussion

CVID is characterized by recurrent sinopulmonary infections, decreased levels of immunoglobulin, and impaired functional antibody responses. The prevalence of CVID is reported to range from one in 50,000 to one in 200,000 [1]. Patients with CVID present with a bimodal age distribution with the majority diagnosed either in childhood or in their second or third decade of life, although some patients present later in their adult life [1]. Autoimmune diseases, including lichen planus [2], are also seen in approximately 25 % of patients with CVID [1].

Cases of corneal disease in patients with CVID are limited in the literature. These studies suggest that keratitis in CVID may manifest as infectious and/or inflammatory in nature [3, 4]. Bilateral consecutive sterile corneal thinning that progressed to perforations was reported in a patient with CVID by Akpek et al. [3]. In this case, multiple scrapings, biopsies, and cultures remained sterile and the corneal infiltrates showed no response to antibiotics but disappeared with topical steroid therapy. It was postulated that the corneal perforations may have initially been due to an autoimmune etiology and then complicated by a secondary *Haemophilus influenzae* endophthalmitis. Though we eventually isolated MRSA from the cornea of our patient, her initial presentation appeared more consistent with a sterile melt (intact epithelium and no infiltrate) and she did not have the typical risk factors for infectious keratitis such as trauma, contact lens wear, or lagophthalmos. Our eventual recovery of MRSA from her cornea and later from her conjunctiva suggests that it is prudent to maintain a high level of suspicion for an infectious etiology in patients with ocular surface disease and CVID even if their initial presentation does not appear to be typical of an infection.

Reports of other ocular manifestations in CVID are also limited and include retinal vasculitis [4], uveitis [4], keratoconjunctivitis [5, 6], and episodic retinal vein occlusions [7]. In several of these cases, initiation of treatment of IVIG and/or steroids led to the resolution of uveitis or granulomatous lesions [4, 5] and recurrent keratoconjunctivitis [5]. Patients that have CVID may require lifelong immunoglobulin replacement to prevent further ocular and systemic manifestations of disease. Corneal cultures may be indicated to evaluate for an infectious etiology, such as in the case presented above. It may be prudent to start patients on empiric topical antibiotics and steroids, given the devastating sequelae seen in the case by Akpek et al. [3].

Common organisms isolated from ocular infections in patients with CVID are *S. aureus* and encapsulated bacteria, such as *Streptococcus pneumoniae* and *H. influenzae*. Ocular infections with these organisms primarily involve the conjunctiva and secondarily involve the cornea. A study by Franklin et al. included five patients with CVID over 2 years. Conjunctival cultures from three patients grew *H. influenzae*, one culture grew *Staphylococcus epidermidis*, and one grew *S. aureus* [8]. Similarly, a patient with multi-organism keratoconjunctivitis described by Ooi et al. had conjunctival cultures that grew *H. influenzae* and *S. aureus* [5]. The patient in the case reported by Akpek et al. eventually developed a secondary endophthalmitis due to *H. influenzae* [3].

Systemic administration of IVIG is the primary treatment for many forms of hypogammaglobulinemia, including CVID. However, it has been demonstrated that even in immunocompetent individuals, levels of immunoglobulins in the tear film do not reflect those in the serum suggesting autonomous local regulation of immunoglobulin [9]. Indeed, it has been established that ocular immunoglobulins are produced locally by both the lacrimal gland and the eye-associated lymphoid tissue in the conjunctiva [10]. Further, IVIG only includes IgG and not other isotypes, such as IgA, the primary immunoglobulin responsible for mucosal defense. Although tear film immunoglobulin levels in patients with CVID have not been studied, it can be conjectured that tear film immunoglobulins are also decreased. Therefore, mucosal defense at the ocular surface could remain susceptible despite systemic IVIG treatment. Further complicating diagnosis and treatment is the fact that while CVID is a form of immunosuppression, it is also associated with autoimmune disease. As noted above, our patient had lichen planus for which she was receiving oral cyclosporine adding to her impaired immune response to corneal infection. We postulate that the combination of CVID and treatment with cyclosporine account for the development and atypical presentation of bacterial keratitis in this patient.

In conclusion, patients with CVID on monthly IVIG infusions may still develop infectious diseases of the ocular surface without the typical findings of bacterial infection. Investigation for an infectious etiology should be performed and treatment promptly implemented when presented with conjunctival or corneal inflammation or ulceration.
